# Antimicrobial Activity of an Amnion-Chorion Membrane to Oral Microbes

**DOI:** 10.1155/2019/1269534

**Published:** 2019-07-11

**Authors:** Haroon Ashraf, Kerri Font, Charles Powell, Michael Schurr

**Affiliations:** ^1^Department of Surgical Dentistry, Division of Periodontics, University of Colorado School of Dental Medicine, Aurora, CO, USA; ^2^Private Practice, Tucson, Arizona, USA; ^3^Department of Immunology and Microbiology, University of Colorado School of Medicine, Aurora, CO, USA

## Abstract

**Objective:**

The aim of this study was to evaluate wound biomodification by assessing antimicrobial properties present within a human-derived composite amnion-chorion membrane (ACM).

**Methods:**

Membranes analyzed were the human-derived ACM BioXclude™ and the porcine-derived collagen membrane Bio-Gide®. Paper discs with and without tetracycline served as positive and negative controls, respectively. The same number of colony-forming units per milliliter for each bacterial species (*Aggregatibacter actinomycetemcomitans*, *Streptococcus mutans*, and *Streptococcus oralis*) was inoculated on each of the discs. Discs from each group were removed at 12 and 24 hours and sonicated to remove the bacteria off the membranes. A serial dilution was performed to quantify bacterial growth.

**Results:**

The ACM inhibited growth at all time points, with all bacterial strains, identical to the negative control tetracycline discs. The collagen membrane and positive controls did not inhibit growth of any of the bacterial species throughout the 24-hour study period. *P* < 0.05 for microbial growth on ACM or negative control vs. either collagen membrane or positive control.

**Conclusion:**

ACM was proven to be as bactericidal as paper discs inoculated with tetracycline at its minimum bactericidal concentration. The ACM bactericidal property may be beneficial in the early wound healing process.

## 1. Introduction

Periodontal disease can lead to intrabony defects, and depending on the size and location, this may lead to the loss of a tooth [1]. Fortunately, advances in periodontal therapy allow for regeneration of the previously lost attachment apparatus in select intrabony defects to improve the overall prognosis [2–4].

There is a wide selection of adjunctive biomaterials that may assist in the regeneration of hard and soft tissue in a surgical wound and enhance the clinical outcome. The process of gaining lost periodontal attachment with the use of membranes has been described as guided tissue regeneration (GTR) [2]. Membranes are biomaterials which provide an effective approach to achieve new attachment within intrabony defects to help with the retention of teeth [5]. Properties within different types of membranes range from ease of handling to the amount of cross-linking and subsequent resorption time [6]. A probable important component within biomaterials is the presence of antimicrobial properties that may increase the efficacy of healing during the initial healing phase.

In surgical procedures, membranes are exposed to microbiota present within the oral cavity during manipulation. It has been shown that in as early as three minutes of intraoral manipulation, there was an average of 10^4^ viable organisms which included those from the red and orange complex [7]. Furthermore, DNA-DNA hybridization assays have shown that after prophylaxis, early colonizers of the streptococci species along with putative periodontal pathogens are present between 0 and 6 hours [8]. The same study identified that species of the red and orange complex and *A. actinomycetemcomitans* were found within the first six hours. Thus, periodontal defects after thorough debridement still have bacteria existing in low levels. These early colonizers play a vital role in the establishment of biofilm formation and can trigger local immunological responses [9].

Guided tissue regeneration membranes which harbor lower levels of microbiota exhibit greater attachment gain [7]. The purpose of this study is to evaluate the presence of antimicrobial properties within a human-derived composite amnion-chorion membrane (ACM) as compared to a collagen membrane that are both used in periodontal therapy.

## 2. Materials and Methods

Analysis of bacterial growth over commercially available membranes was conducted in three separate *in vitro* trials. Test membranes included a human-derived amnion-chorion membrane (BioXclude®, Snoasis Medical, Golden, CO) and a porcine-derived collagen membrane (Bio-Gide®, Geistlich, Princeton, NJ). Blank paper discs (BBL™, Sparks, MD) measuring 6 mm in diameter were used in the study as a negative control. The same paper discs containing tetracycline were used as a positive control at a bactericidal concentration of 62 *μ*g/mL. Three bacterial species were used in the study. These included *Aggregatibacter actinomycetemcomitans* ATCC 33384, *Streptococcus mutans* ATCC 25175, and *Streptococcus oralis* ATCC 9811.

**Figure 1:**
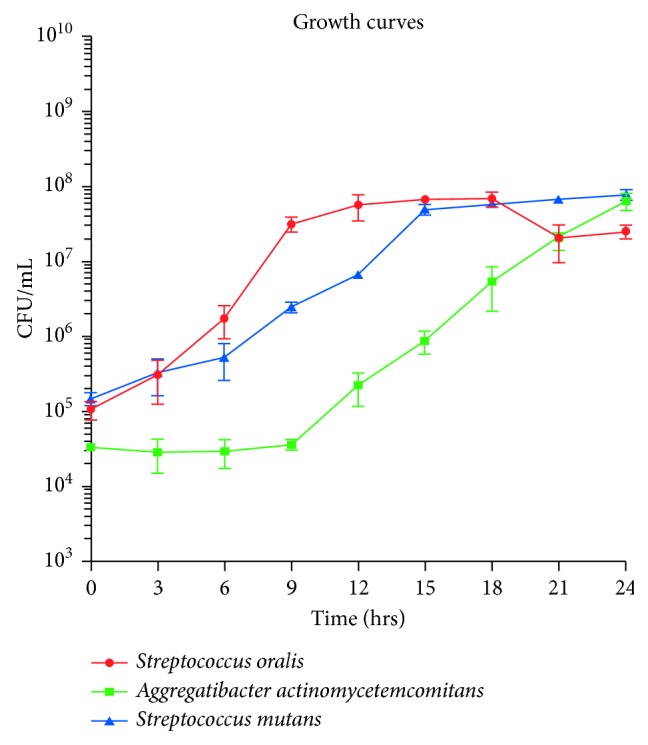
Growth curves for each bacterial species used in the study.

Growth curves were established for each bacterial species to assess viability and determine the time parameters of the experiment (Figure 1).

Cells were seen to be viable within a 24-hour time frame. Bacterial species were isolated and grown individually on brain heart infusion (BHI) agar medium (BBL™, Sparks, MD) under optimal conditions in an incubator (Forma Scientific™, Marietta, OH) set at 37°C and 5% CO_2_ for 24 hours. Isolated bacterial colonies were then transferred aseptically into sterile 14 mL polypropylene tubes (Falcon®, Tewksbury, MA) containing 5 mL liquid BHI medium and labeled appropriately. The samples were left to incubate until the cells were most viable before entering their stationary phase respective to their growth curves. At the proper time point, 2 *μ*l of the bacteria suspended in the previous liquid medium was transferred into another 14 mL polypropylene tube containing 2 mL liquid BHI medium. The concentration of this inoculum was recorded as time “0” by means of performing a serial dilution from the liquid sample. The test and control discs were placed on separate BHI agar plates for a total of four test groups. The test discs were cut into 6 mm diameter circular discs in sterile conditions (same diameter as control discs), and 10 *μ*l of the initial inoculum was placed onto each disc. The same number of CFU/mL for the respective bacterial species was inoculated on each membrane and control discs at the beginning of the 24-hour trials on separate discs.

**Figure 2:**
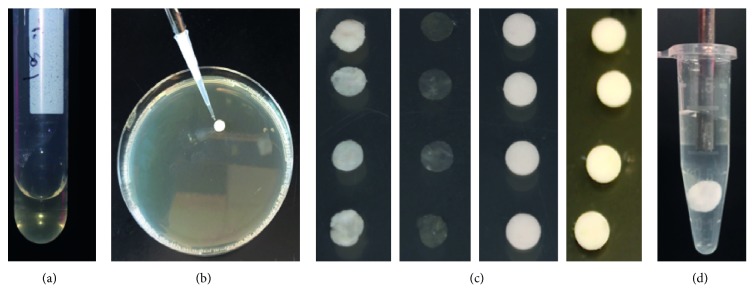
(a) Example of initial inoculum of isolated *S.o.* in BHI medium. (b) Inoculation of bacteria onto collagen disc. (c) Example of inoculated discs ready for the incubation period (collagen membrane, ACM, negative control, and positive TCN control from left to right). (d) Sonication of bacteria from disc into PBS solution.

At 12 and 24 hours, the discs from each test group were removed and transferred into 1.5 mL centrifuge tubes (Fisher Scientific, Pittsburgh, PA) containing 1 mL of phosphate-buffered saline (PBS). The samples were then sonicated with an ultrasonic probe (Fisher Scientific, Pittsburgh, PA) at 1 watt for 15 seconds to agitate the bacteria off the membranes into the PBS solution. A serial dilution was performed on the buffer solution containing the bacteria to quantify growth (Figures 2(a)–2(d)).

**Figure 3:**
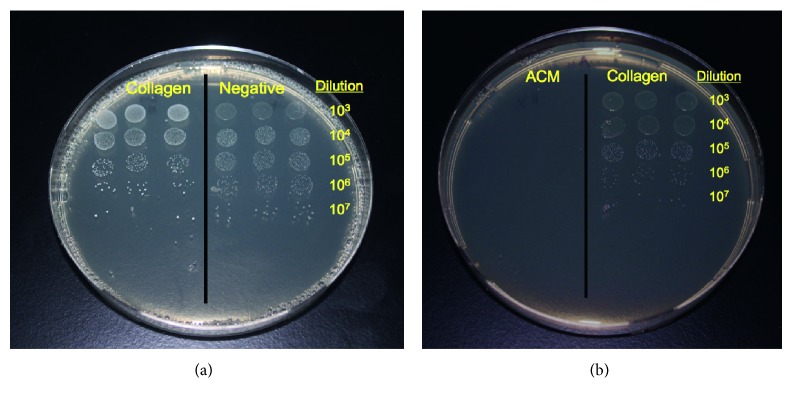
(a) Bacterial growth following removal of attempted *S.o.* culture from the collagen membrane (left half of the plate) and negative control (right half of the plate) at 24 hours following serial dilution. (b) Bacterial growth following removal of attempted *A.a.* culture from the ACM (left half of the plate, including plating of “inoculum”) and collagen membrane (right half of the plate) at 24 hours following serial dilution.

The plating of the serial dilution for ACM and positive control discs included a direct placement of the PBS solution from the centrifuge tube that contained any sonicated bacteria from the samples (Figures 3(a) and 3(b)). A total of three separate *in vitro* trials were performed in triplicate in the above manner. This resulted in a total of seventy-two discs inoculated in each trial for all three bacterial species.

## 3. Data Collection

**Figure 4:**
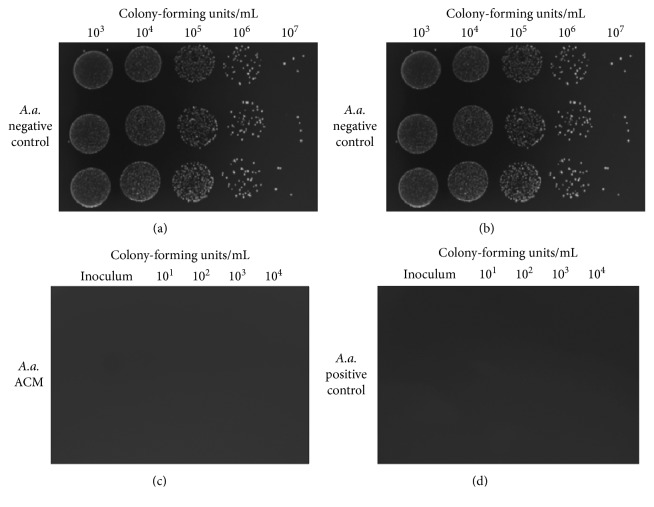
High contrast image of CFU/mL for *A.a.* culture during a trial on (a) collagen membrane; (b) negative control; (c) ACM; (d) positive control.

A serial dilution was performed from the sonicated solution to quantify bacterial growth by counting the colony-forming units per milliliter (CFU/mL) present. The colonies were counted at time “0” prior to inoculation, at 12 hours and at 24 hours. Depending on the presence of quantifiable colonies, the number of bacteria was averaged until each column in the row of triplicate growth had a value greater than zero for a fair and unbiased quantification. The average of each column consisted of its own data point. Considering each of the discs was plated in triplicate, there were three columns of data per disc. This resulted in a total of 216 data points entered into an Excel spreadsheet for the 12- and 24-hour growths. Additionally, the initial inoculum concentration prior to placement on the membranes was recorded for each trial (Figures 4(a)–4(d)).

## 4. Statistical Methods

The median microbial counts and the interquartile ranges of the three bacterial species on each membrane surface were calculated at times 0, 12, and 24 hours. A Wilcoxon signed rank test was performed to compare the differences in microbial growth. This is a paired difference test allowing comparison of two sets of measurements to assess whether population means differ. Statistical analysis was carried out by using RStudo v0.99.484 software. A type 1 error value of 0.05 was used to account for any statistically significant differences.

The statistical analysis was supported by NIH/NCATS Colorado CTSI Grant Number UL1 TR001082. Contents are the authors' sole responsibility and do not necessarily represent official NIH views.

## 5. Results

A total of two-hundred sixteen data points for the 12- and 24-hour time points were recorded after three trials were completed. With these data, median microbial counts could be calculated for each species on each membrane at their respective times (Table 1).

After 12 hours of incubation, the collagen membrane and negative control discs had significantly greater numbers of bacterial growth than the ACM across all bacteria types. For *A. actinomycetemcomitans*, median microbial counts at 12 hours on the collagen membrane were 4.2 × 10^5^ CFU/mL (range: 2.95 × 10^5^ to 4.05 × 10^6^) and on negative control discs were 2.0 × 10^5^ CFU/mL (range: 1.3 × 10^5^ to 3.4 × 10^6^). For *S. mutans*, median microbial counts at 12 hours on the collagen membrane were 5.0 × 10^6^ CFU/mL (range: 1.7 × 10^5^ to 8.0 × 10^6^) and on negative control discs were 4.4 × 10^6^ CFU/mL (range: 3.8 × 10^5^ to 6.0 × 10^6^). For *S. oralis*, median microbial counts at 12 hours on the collagen membrane were 7.0 × 10^6^ CFU/mL (range: 6.4 × 10^6^ to 8.0 × 10^6^) and on negative control discs were 5.75 × 10^6^ CFU/mL (range: 3.5 × 10^6^ to 1.0 × 10^7^). The median microbial counts on the ACM and TCN-treated positive control discs were zero for each bacterial species at 12 hours.

After 24 hours of incubation, the collagen membrane and negative control discs had significantly greater numbers of bacterial growth than the ACM across all bacteria types. For *A. actinomycetemcomitans*, median microbial counts at 24 hours on the collagen membrane were 1.35 × 10^7^ CFU/mL (range: 1.25 × 10^6^ to 1.0 × 10^8^) and on negative control discs were 1.4 × 10^7^ CFU/mL (range: 1.0 × 10^7^ to 1.9 × 10^7^). For *S. mutans*, median microbial counts at 24 hours on the collagen membrane were 7.37 × 10^7^ CFU/mL (range: 1.3 × 10^7^ to 6.1 × 10^8^) and on positive negative discs were 1.35 × 10^7^ CFU/mL (range: 1.0 × 10^7^ to 1.8 × 10^7^). For *S. oralis*, median microbial counts at 24 hours on the collagen membrane were 2.2 × 10^7^ CFU/mL (range: 1.5 × 10^7^ to 2.8 × 10^7^) and on negative control discs were 6.0 × 10^7^ CFU/mL (range: 3.5 × 10^7^ to 7.0 × 10^7^). The median microbial counts on the ACM and TCN-treated positive control discs were zero for each bacterial species at 24 hours.

**Figure 5:**
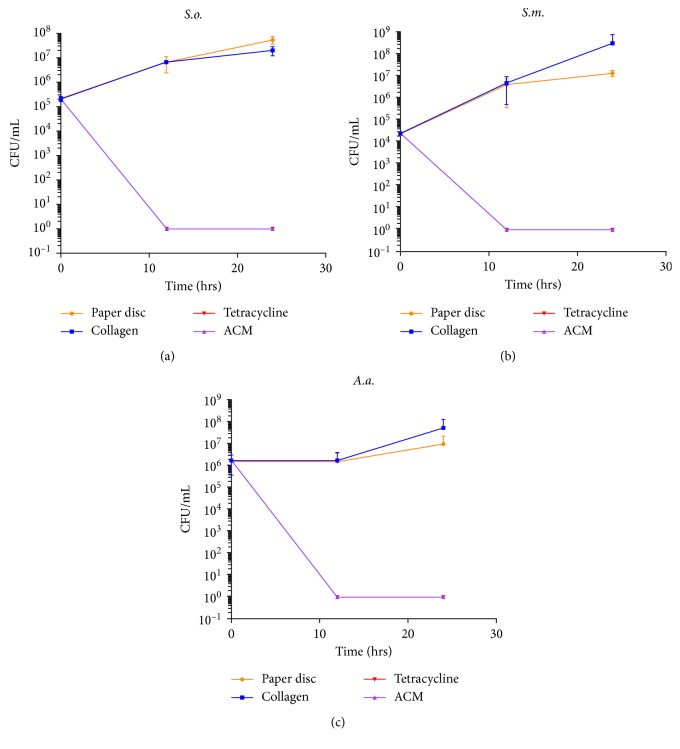
Kill curve for each bacteria on each test and control disc: (a) *S.o.*; (b) *S.m.*; (c) *A.a.* Note: tetracycline and ACM curve are superimposed on each other.

With the initial concentration of bacteria inoculated onto the test and control discs, kill curves could be computed to show the pattern of growth or death of the bacterial species on the test and control discs. This was plotted for each species as a function of CFU/mL vs. time (Figure 5).

## 6. Discussion

The findings from this study indicate that the ACM was as bactericidal as the TCN-treated positive control. The collagen membrane did not demonstrate any antimicrobial properties and facilitated growth of these species. Tetracycline was chosen as it has been shown to be effective in the management of periodontal diseases. It is a broad-spectrum antimicrobial agent that is primarily bacteriostatic and highly effective against Gram-negative bacterial species. Additional benefits of this antibiotic class include the ability to bind fibroblasts, inhibit collagenase, downregulate osteoclasts, and decrease anti-inflammatory mediators [10].

The addition of a local antibiotic at the time of regenerative surgery may not allow for substantivity of the antibiotic. The crevicular flow rate within the sulcus can be as high as 137 *μ*l/h in patients with advanced periodontal disease [11]. Oral administration of TCN is seen to increase levels of gingival crevicular fluid (GCF) to 3–10 *μ*g/mL after 48 hours [12]. The effect of TCN at these concentrations in GCF is lower than the effect the ACM had as compared to the positive control concentration, which adds to the significance of our findings.

Bacterial presence along a surgically approximated wound will enter through the incision line that communicates with the oral cavity. This may be either intrasulcular incision adjacent to the tooth surface [13] or over a linear crestal incision to access the defect. Following surgical incision of the previous attachment on the tooth surface, the junctional epithelium begins to re-establish itself. The junctional epithelium originates from the adjacent oral epithelium and is seen to form new attachment in as little as five days [14]. Thus, if the ACM is able to exert its bactericidal effect during this time of the wound exposure to the outside microbiota, this could be beneficial for better regeneration outcomes as compared to a site where bacterial pathogens were present. This has been demonstrated clinically in both GTR and ridge preservation. GTR demonstrated greater attachment at re-evaluation in membranes with decreased microbiota presence [7]. A decreased inflammatory response within the first week of ridge preservation demonstrated improved bone gain [15]. It is known that early exposure of membranes used in GTR is detrimental to achieving maximal attachment gain [16, 17]. Colonization of bacteria on membranes may lead to later exposure, or this may occur as a secondary consequence due to membrane exposure [7].

The collagen membrane used in the study appeared not to have any antimicrobial properties. The use of such membranes in sites of GTR may allow for an increased amount of microbiota interfering with the regenerative potential. Exposed membranes within the oral cavity would be subjected to the presence of multiple bacteria, including *A. actinomycetemcomitans*, *F. nucleatum*, *P. gingivalis*, *P. intermedia*, and *S. mutans*. In fact, the concentration of the above bacterial species increased over time [18].

Local bacterial insults can trigger immune responses that alter the desired treatment outcome in regeneration. Toxins produced by microbes such as lipopolysaccharides or phagocytosis of bacteria can trigger macrophages to initiate an inflammatory response. This response includes an increase in leukocyte infiltrate composed of neutrophils and polymorphonuclear leukocytes [19]. Neutrophils along with B cells and T cells can activate receptor activator of nuclear factor kappa *β* ligand (RANKL) and upregulate receptor activator of nuclear factor kappa *β* (RANK) leading to an increased level of osteoclastic activity [20].

Current evidence that supports the findings of this study has shown an inhibitory effect from fetal chorioamniotic membranes against a range of bacteria. The aforementioned membranes were freshly separated from women undergoing cesarean section to be placed onto agar plates with a 10^9^ per mL bacterial suspension [21]. This study shows that the antibacterial properties are still present even after the extensive tissue engineering process used to prepare ACM for clinical use. Furthermore, other studies have shown that the amniotic membrane can suppress IL-1*α* and Il-1*β* that are upregulated through LPS [22]. The aforementioned presence of an inhibitor of MMPs may decrease the collagen destruction that is undesirable in periodontal regeneration. Despite the specific anti-inflammatory immunologic mechanisms, the most important property of the ACM may be the antibacterial components that prove to be present within the processed and dehydrated ACM.

It is important to determine the antibacterial components within the ACM. One possible source of the antibacterial properties may be beta-defensins produced by cells. Beta-defensins are a family of antimicrobial peptides that resist microbial colonization of epithelial surfaces. It has been shown that beta-defensins are present throughout ACM. For instance, experiments utilizing reverse transcription polymerase chain reaction (RT-PCR) with amnion epithelial cells have expressed mRNA for all beta-defensins 1–4, with significantly higher levels of *β*3 [23]. Other studies have hinted the presence of secretory leukocyte protease inhibitors and elafin within the lining of placental tissues having antimicrobial properties [24]. Both beta-defensins and elafin are seen in the chorion trophoblast layers of fetal membranes and placenta [25].

Limitations of the current study are noted by not demonstrating the bactericidal properties within a biofilm or using a longer study period. Both of these factors were considered, but the complex nature of biofilms and the limitations from the natural growth curves before they entered their death phase would not allow for accurate assessment of decreased cell viability due to the test samples or some other outside variables.

## 7. Conclusion

It was determined that the ACM was as bactericidal as the positive control paper discs treated with TCN at a bactericidal concentration. The collagen membrane does not appear to have antimicrobial properties due to its support of the bacterial growth similar to the negative control discs. The findings from this study are valuable to the clinician when selecting a membrane for regenerative procedures to enhance surgical outcomes.

## Figures and Tables

**Table 1:** Median microbial counts across trials.

Species and initial inoculation (CFU/mL)	Time (hours)	Collagen (CFU/mL)	ACM (CFU/mL)	Negative control (CFU/mL)	Positive control TCN (CFU/mL)
*A.a.* 1.8 × 10^6^	12	4.25 × 10^5^ (2.95 × 10^5^–4.05 × 10^6^)	0^*∗*^	2.00 × 10^5^ (1.30 × 10^5^–3.40 × 10^6^)	0^*∗*^
	24	1.35 × 10^7^ (1.25 × 10^6^–1.00 × 10^8^)	0^*∗*^	1.40 × 10^7^ (1.00 × 10^7^–1.90 × 10^7^)	0^*∗*^

*S.m.* 2.4 × 10^4^	12	5.00 × 10^6^ (1.70 × 10^5^–8.00 × 10^6^)	0^*∗*^	4.40 × 10^6^ (3.80 × 10^5^–6.00 × 10^6^)	0^*∗*^
	24	7.37 × 10^7^ (1.30 × 10^7^–6.10 × 10^8^)	0^*∗*^	1.35 × 10^7^ (1.00 × 10^7^–1.80 × 10^7^)	0^*∗*^

*S.o.* 2.3 × 10^5^	12	7.00 × 10^6^ (6.40 × 10^6^–8.00 × 10^6^)	0^*∗*^	5.75 × 10^6^ (3.50 × 10^6^–1.00 × 10^7^)	0^*∗*^
	24	2.20 × 10^7^ (1.50 × 10^7^–2.80 × 10^7^)	0^*∗*^	6.00 × 10^7^ (3.50 × 10^7^–7.00 × 10^7^)	0^*∗*^

^*∗*^ *P* < 0.05 for microbial growth on ACM or positive control vs. either collagen membrane or negative control.

## Data Availability

The discrete quantitative data used to support the findings of this study are included within the article.
